# Comorbid depressive symptoms can aggravate the functional changes of the pain matrix in patients with chronic back pain: A resting-state fMRI study

**DOI:** 10.3389/fnagi.2022.935242

**Published:** 2022-07-18

**Authors:** Guangfang Zhang, Junqin Ma, Weirong Lu, Hongrui Zhan, Xuefei Zhang, Kangling Wang, Yingxuan Hu, Xianglong Wang, Weiwei Peng, Shouwei Yue, Qingxiang Cai, Wen Liang, Wen Wu

**Affiliations:** ^1^Department of Rehabilitation Medicine, Zhujiang Hospital, Southern Medical University, Guangzhou, China; ^2^Department of Pain, Guangdong Provincial Hospital of Chinese Medicine, Guangzhou, China; ^3^Department of Radiology, Zhujiang Hospital, Southern Medical University, Guangzhou, China; ^4^Department of Physical Medicine and Rehabilitation, The Fifth Affiliated Hospital of Sun Yat-sen University, Zhuhai, China; ^5^School of Psychology, Shenzhen University, Shenzhen, China; ^6^Department of Rehabilitation Medicine, Qilu Hospital of Shandong University, Jinan, China; ^7^Department of Anesthesiology, The First Affiliated Hospital of Guangzhou University of Chinese Medicine, Guangzhou, China

**Keywords:** comorbid depressive symptoms, chronic back pain, pain matrix, fMRI, resting-state

## Abstract

**Objective:**

The purposes of this study are to explore (1) whether comorbid depressive symptoms in patients with chronic back pain (CBP) affect the pain matrix. And (2) whether the interaction of depression and CBP exacerbates impaired brain function.

**Methods:**

Thirty-two patients with CBP without comorbid depressive symptoms and thirty patients with CBP with comorbid depressive symptoms were recruited. All subjects underwent functional magnetic resonance imaging (fMRI) scans. The graph theory analysis, mediation analysis, and functional connectivity (FC) analysis were included in this study. All subjects received the detection of clinical depressive symptoms and pain-related manifestations.

**Result:**

Compared with the CBP group, subjects in the CBP with comorbid depressive symptoms (CBP-D) group had significantly increased FC in the left medial prefrontal cortex and several parietal cortical regions. The results of the graph theory analyses showed that the area under the curve of small-world property (*t* = −2.175, *p* = 0.034), gamma (*t* = −2.332, *p* = 0.023), and local efficiency (*t* = −2.461, *p* = 0.017) in the CBP-D group were significantly lower. The nodal efficiency in the ventral posterior insula (VPI) (*t* = −3.581, *p* = 0.0007), and the network efficiency values (*t* = −2.758, *p* = 0.008) in the pain matrix were significantly lower in the CBP-D group. Both the topological properties and the FC values of these brain regions were significantly correlated with self-rating depression scale (SDS) scores (all FDR corrected) but not with pain intensity. Further mediation analyses demonstrated that pain intensity had a mediating effect on the relationship between SDS scores and Pain Disability Index scores. Likewise, the SDS scores mediated the relationship between pain intensity and PDI scores.

**Conclusion:**

Our study found that comorbid depressive symptoms can aggravate the impairment of pain matrix function of CBP, but this impairment cannot directly lead to the increase of pain intensity, which may be because some brain regions of the pain matrix are the common neural basis of depression and CBP.

## Introduction

Chronic pain is a debilitating disease that affects physiology and psychology. The World Health Organization’s large-scale survey found that the prevalence of chronic pain is very high in both developed (37%) and developing (41%) countries (Tsang et al., 2008), so it greatly increases the cost of medical resources (Henschke et al., 2015). A growing body of literature (Velly and Mohit, 2018) shows that individuals with chronic pain also have a sharply higher risk of developing psychological disorders [Gureje et al. (1998) found that this risk was four times higher in individuals without chronic pain]. However, previous medical views often regarded the patient’s body and psychology as independent and separate structures (Gatchel et al., 2007). In the process of diagnosis and treatment of patients with chronic pain, physicians generally pay more attention to the physical complaints of patients with chronic pain and relatively ignore the impaired psychological and social functions of patients.

There is strong evidence that pain is a major risk factor for depression and that depressive symptoms aggravate the progression of chronic pain (Tappe-Theodor and Kuner, 2019). When patients with chronic pain have comorbid depressive symptoms, this comorbidity condition can make the two have mutual promotion of some distresses, such as poor functionality and higher disability rates (Baumeister et al., 2011; Hall et al., 2011). Unfortunately, the underlying mechanism of this reciprocal interaction is unclear yet and needs to be taken seriously.

Some previous preclinical and clinical studies have already paid some attention to the potential neural mechanism of the interaction between depression and chronic pain. For example, a preclinical study has identified a novel neural pathway from the dorsal raphe nucleus (involving 5-hydroxytryptamine projections) to the central nucleus of the amygdala (somatostatin-expressing neurons) that not only mediates depressive symptoms, but also affects painful symptoms (Zhou et al., 2019). A neuroimaging study found that functional connectivity (FC) between the thalamus and dorsolateral prefrontal cortex (involved in the pain descending regulation system) modulated the relationship between depression and chronic low back pain (Li, 2021). A study on heat pain sensitivity found that heat pain sensitivity was associated with thalamic, amygdala, cingulate cortex and sensory cortex activity in both chronic low back pain patients and depressed patients (Rodriguez-Raecke et al., 2014). Moreover, depression-related neuroimaging studies have shown that depression leads to altered FC between a number of brain regions, including the anterior cingulate cortex-thalamus, the anterior cingulate cortex-insula, and the prefrontal-limbic-thalamic circuit, which partially overlap with descending pain modulatory pathway (Gong and He, 2015). These studies suggest that chronic pain and depression have some common neural circuits and partially overlapping functional networks. However, although taking steps in the right direction, the neurological study of depression and pain co-occurrence is still in a preliminary and approximate scope, and there is much room for extending the underlying neural mechanisms of the interaction of chronic pain and depression.

Over the past decade, a great deal of studies have been devoted to understanding how the brain responds to pain and have already demonstrated altered brain structure and function in patients with acute and chronic pain (Apkarian et al., 2004; Ichesco et al., 2014; Lieberman et al., 2014). These wide-ranging cortical and subcortical brain areas, including primarily the thalamus, insula cortex, sensory cortex, and anterior cingulate cortex (ACC), are known as the pain matrix (Legrain, 2011; Geuter et al., 2020). Therefore, since the underlying neural mechanisms by which chronic pain and depression interact are not fully understood, the pain matrix, a core network of pain processing, should deserve focused attention. In addition, little is known about whether depression and chronic pain interact to exacerbate alterations in brain function or structure, which is one of the concerns we are interested in.

Based on the above description, resting-state functional magnetic resonance imaging (fMRI), a recognized objective and non-invasive measure of brain activity, has also been used as a measure of potential biomarkers for detecting various types of pain (Hohenfeld et al., 2018), and was used in this study to explore the changes in brain functions of subjects. Subjects with chronic back pain (CBP) were included in this study and were divided into a group with comorbid depressive symptoms and a group without depressive symptoms. Graph theory analysis methods and FC analysis methods were used in this study to explore the differences in brain function between groups. The self-rating depression scale (SDS) (Zung, 1965) was used to measure the psychological status of the subjects. The Pain Disability Index (PDI) (Pollard, 1984) was used to measure the degree to which patients’ daily lives are disrupted by chronic pain. The main objectives of this study are: (1) to explore whether the comorbid depressive symptoms can affect the pain matrix (the core brain network for pain), and (2) to determine whether the interaction between the comorbid depressive symptoms and CBP exacerbate the impairment of brain function. We hypothesized that comorbid depressive symptoms can lead to altered pain matrix function in patients with chronic pain, which in turn affects patients’ pain symptoms.

## Materials and methods

### Participants

To control the covariates, all the subjects were patients with chronic musculoskeletal back pain. Patients with a SDS score ≥ 50 were considered to have comorbid depressive symptoms (Zung, 1967; Edelstein et al., 2010). All subjects in this study were able to understand all items on the SDS scale and completed the SDS scale. We recruited 30 patients with comorbid depressive symptoms (CBP-D) and 32 patients without comorbid depressive symptoms (CBP) from Zhujiang Hospital of Southern Medical University and Guangdong Provincial Hospital of Chinese medicine. Two subjects in the CBP group were excluded from this study due to image quality problems or head movement factors. Finally, 30 patients with comorbid depressive symptoms (CBP-D) and 30 patients without comorbid depressive symptoms (CBP) were recruited. The inclusion criteria were: (1) the visual analog scale (VAS) score of pain intensity was greater than or equal to 3 points and the duration was more than 3 months; (2) those who were assessed as normal or abnormal by SDS; (3) no fMRI contraindication; (4) did not receive psychological induction training; and (5) without cerebral lesions. The exclusion criteria were: (1) subjects with life-threatening primary diseases such as those of the cardio-cerebrovascular, liver, kidney, or hematopoietic system; (2) subjects with psychiatric diseases, tuberculosis, tumor, rheumatism, active gastrointestinal ulcers or asthma; (3) subjects with irregular menstrual cycles or currently menstruating; (4) subjects with spinal fracture, dislocation, acute cervical or lumbar disk herniation; (5) subjects with magnetic component objects in their bodies; and (6) body mass index greater than 25 kg/m^2^. This experiment was approved by the ethics committee of Zhujiang Hospital of Southern Medical University and Guangdong Provincial Hospital of Chinese medicine. The clinical registration number is ChiCTR2200056929. The research purpose of this manuscript is a part of the whole clinical research project. All participants signed informed consent. We explained the detailed instructions, experimental procedures, possible risks, and discomforts of the study to all subjects, as well as answered their questions in detail.

### Data acquisition

All image data were collected by a Philips 3.0 T Ingenia magnetic resonance imager in Zhujiang Hospital, Southern Medical University, and scanned in a standard radio-frequency head coil. Structure data were acquired with a high resolution 3D T1-weighted sequence: repetition time (TR)/echo time (TE) = 7.2/3.3 ms; flip angle = 7°; field of view (FOV) = 256 mm × 256 mm; matrix size = 256 × 256; 1 mm × 1 mm in-plane resolution; slice thickness = 1 mm; 176 slices; slice gap = 0 mm. Functional MRI data were acquired using a T2^∗^-weighted, single-shot, gradient-recalled echo planar imaging sequence, TR/TE = 2000/30 ms; field of view (FOV) = 224 mm × 224 mm; matrix size = 64 × 63; flip angle = 90°; 3.5 mm × 3.5 mm in-plane resolution; slice thickness = 3.5 mm; 33 slices; slice gap = 0.7 mm; number of signals averaged (NSA) = 1.

### Measures

The PDI is a self-rating scale that contains seven items that can evaluate the extent to which pain affects seven categories of life activity. Each item is scored on a scale of 0 to 10, with 0 representing no pain induced disability at all and 10 representing that daily life has been totally disrupted by the pain. The SDS is a 20-item self-assessment scale that is easy to use and visually reflects the patient’s subjective feelings. Each item is divided into 4 levels: no or little time, little time, quite a lot of time, and most of the time or all the time. The level of each item refers to the frequency with which the subject experiences the item.

### Data preprocessing

The fMRI data preprocessing was carried out in the DPABI toolbox (Yan et al., 2016) based on Statistical Parametric Mapping (SPM12) software^1^. This procedure included data format conversion, discarding the first 10 volumes, slice timing correction, realignment, nuisance covariates regressors, spatial normalization, smoothing, linear de-trending, and filtering (see Supplementary Material for the detail). The demographic data (such as age, sex ratio, and pain duration) with significant differences between the two groups were also included in linear regression as covariates.

### Functional data analysis

Numerous studies have shown that acute or chronic pain causes alterations in several cortical or subcortical structures, which are collectively referred to as the pain matrix (Legrain et al., 2011; Geuter et al., 2020). Therefore, based on the purpose of our study, the various brain regions of the pain matrix were prior defined as seeds and were extracted as a mask (see Supplementary Table 1 for the region information of the seeds) using the Human Brainnetome Atlas (Fan et al., 2016). The seeds-to-whole-brain FC analysis and the Fisher’s *r*-to-*z* transform were then calculated successively. Multiple comparison correction based on Gaussian random field theory (GRF) correction (voxel-level forming statistical threshold of *z* = 2.58, cluster-level *p* < 0.05). After the correction, the results of the seeds-to-whole-brain (significant seeds labeled as 155, 160, and 162 in the Human Brainnetome Atlas, see Supplementary Table 1 for the region information of regions of interest) were included in the follow-up analysis. The seeds-to-whole-brain results together with the significant seeds were extracted as regions of interest (ROIs, Supplementary Figure 1) and calculated for ROI-ROI FC analysis. Finally, an *n*^∗^*n* correlation matrix (*n* is the number of ROIs) was generated for each subject and the statistical analysis of the inter group was carried out (*q* = 0.05, false discovery rate corrected). The FC map and the correlation coefficient between the significant brain areas were shown by the heat map of the FC correlation coefficient and BrainNet Viewer (Xia et al., 2013).

### Graph theory analyses

The graph theory is a mathematical system to study networks. The brain network can be defined as a graph by *G* (*N*, *K*), with *N* representing the number of nodes (brain regions) and *K* representing the number of edges (functional or structural connectivity) (Wang et al., 2010). In the current study, we calculated various topological properties to evaluate brain activity. The Human Brainnetome Atlas with 246 brain ROIs was used in the graph theory analysis. The brain was first parcellated into 246 regions according to the template. Pearson correlation of the time series between each brain region was then calculated and generated a 246^∗^246 correlation matrix for each subject. Setting different thresholds will lead to various results, and there is no threshold gold standard at present (Garrison et al., 2015). To avert weak and spurious links in the functional network, the ideal way is to set the threshold to a wider range, then calculate the topological metrics under each threshold, and get the area under the curve (AUC) (Rubinov and Sporns, 2010). In this work, we applied the sparsity threshold from 3 to 30% in steps of 1% into a binary graph (calculated based on the built-in code of the GRETNA toolbox).

We computed five main topological metrics (i.e., global efficiency, local efficiency, nodal efficiency, nodal local efficiency, and small-world property) by using the GRETNA toolbox^2^ (see Supplementary Material for the detailed definition and the interpretation of equations). To find whether there was any difference in the activity of the key brain regions (pain matrix) between the two groups. We also extracted the nodal efficiency and the nodal local efficiency of all nodes in the pain matrix, as well as calculated the network efficiency and local efficiency of the pain matrix for each participant, and the inter-group comparisons of the topological properties were also carried out (*q* = 0.05, false discovery rate corrected).

### Correlation analyses and mediation analyses

The Pearson correlation analyses were used to calculate the correlation between the neuroimaging data with the clinical characteristics results. Spearman correlation analyses were used for correlation analysis of non-normally distributed data. The boxplot method was used to identify outliers of the clinical characteristics and then eliminate them. To further test the summary of the relationships among the pain, the comorbid depressive symptoms, and the neuroimaging findings, mediation analyses were conducted based on the results of correlation analyses. In this study, we designed two main mediating models, in which the mediating variable (M) in model 1 was the ROI-ROI FC, and the purpose of designing this model was to analyze whether FC could mediate the relationship between pain and comorbid depressive symptoms. Model 2 made pain and comorbid depressive symptoms mutually mediating variables, which was designed to analyze whether the reciprocal interaction between depressive symptoms and pain would affect the clinical characteristics or neuroimaging findings of patients. The mediation analyses were performed using the PROCESS macro for SPSS^3^ (Preacher and Hayes, 2004; Williams and Mackinnon, 2008). The bootstrap samples were *n* = 5,000, and significant indirect effects were defined by a 95% confidence interval (CI) that does not include zero.

### Statistical analysis

The Chi-squared and two-independent sample *t*-tests were employed to conduct the group differences in demographic data by SPSS22.0 (SPSS, Chicago, IL, United States) software. The Shapiro–Wilk test was used to assess the normality of distribution of the data. The Mann–Whitney *U* test was used to compare non-normal distribution data. Analysis of covariance was used to determine whether the difference in pain intensity between groups affected the difference in FC results between groups. All statistical assessments were two-tailed, and the significance threshold was *p* = 0.05.

## Results

### Demographic and clinical characteristics results

There were no significant differences in age, sex ratio, or pain duration between the two groups (Table 1). Compared with the CBP group, the CBP-D group had significantly higher SDS (*t* = 10.360, *p* < 0.001) scores, self-report pain intensity (*Z* = 3.661, *p* < 0.001), and PDI scores (*t* = 4.705, *p* < 0.001) (Table 1).

**TABLE 1:** Demographic and clinical characteristics and behavioral scores of the participants.

	CBP-D (*n* = 30)	CBP (*n* = 32)	Test statistics	*P*-value
**Demographic features**				
Age, years	40.30 ± 15.39	38.31 ± 14.54	0.523	0.603
Sex ratio, female	21 (70%)	20 (62.5%)	0.398*	0.533
Pain duration, months	24 (45)	18 (28.42)	−0.459**	0.646
**Pain symptom severity**				
Pain intensity, by VAS	5 (2)	4 (2)	3.661**	<0.001
PDI scores, by VAS	36.63 ± 12.58	20.97 ± 13.57	4.705	<0.001
SDS rating	59.04 ± 7.99	39.14 ± 7.13	10.360	<0.001

**Mann–Whitney *U* test. *Chi-square test. The results satisfying normal distribution were expressed as means ± standard deviations, otherwise were expressed as median (inter-quartile range).

The correlation analysis results showed that the SDS scores were significantly positively correlated with pain intensity (*r* = 0.4387, *p*-FDR < 0.05, *p*-uncorrected = 0.0004) and PDI scores (*r* = 0.4811, *p*-FDR < 0.05, *p*-uncorrected < 0.0001). Pain intensity was also significantly positively correlated with PDI scores (*r* = 0.7842, *p*-FDR < 0.05, *p*-uncorrected < 0.0001) (Supplementary Figure 2).

### Neuroimaging results

**FIGURE 1:**
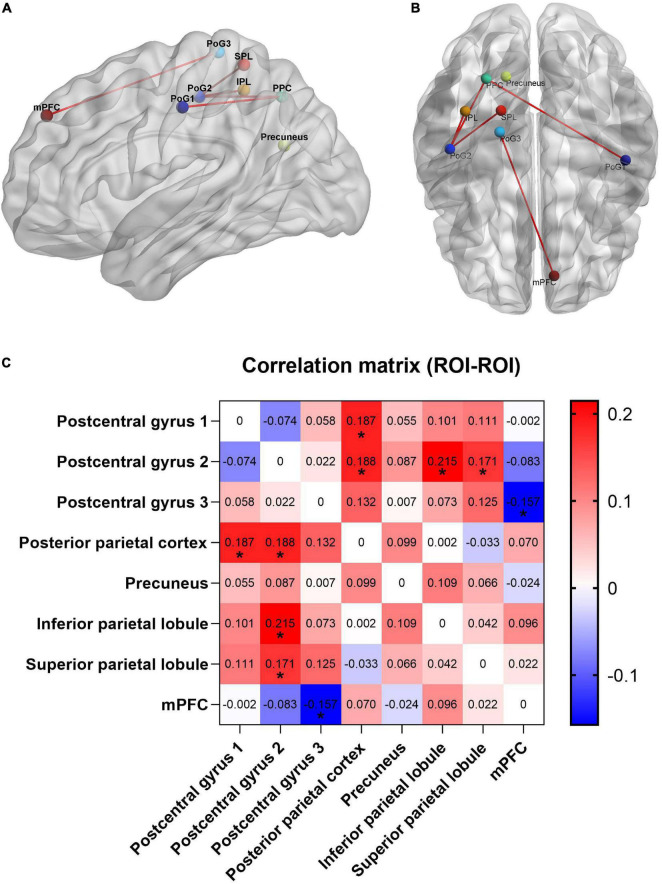
Functional connectivity results with significant difference between groups. **(A)** Sagittal view. **(B)** Axial view. **(C)** Heat map of functional connectivity between groups from ROI–ROI analysis. The parameters represent the FC correlation coefficient. *Represents the functional connectivity of brain regions with significant correlation after FDR correction. PPC, posterior parietal cortex; PoG, postcentral gyrus; mPFC, medial prefrontal cortex; SPL, superior parietal lobule; IPL, inferior parietal lobule.

Compared with the CBP group, subjects in the CBP-D group had significantly increased right postcentral gyrus (PG1, area 2) FC to the right posterior parietal cortex (PPC), right inferior parietal lobule (IPL), and right superior parietal lobule (SPL). The CBP-D group also showed significantly increased left postcentral gyrus (PG2, area 1/2/3) FC to the right PPC, and significantly decreased right postcentral gyrus (area 1/2/3, trunk region) FC to the left medial prefrontal cortex (mPFC) (Figure 1 and Table 2).

**TABLE 2:** The brain regions with significant functional connectivity between groups.

Regions	R/L	BA	Cluster size voxels	MNI			*z*-values
					
				*x*-	*y*-	*z*-	
Posterior parietal cortex	R	7	215	27	−63	48	3.6239
Precuneus	R	7	215	27	−63	48	3.6239
Inferior parietal lobule	R	7	158	39	−45	51	4.2427
Superior parietal lobule	R	5, 7	158	39	−45	51	4.2427
mPFC	L	9	147	−9	48	39	−3.9663

BA, Brodmann Area. MNI, Montreal Neurological Institute. R/L, right or left. mPFC, medial prefrontal cortex.

**FIGURE 2:**
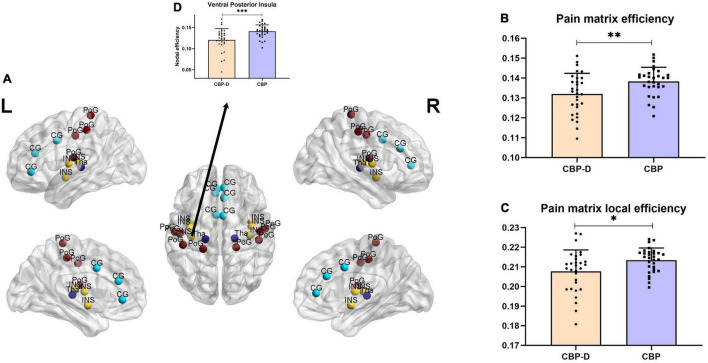
Inter group comparison of topological properties of pain matrix. **(A)** Distribution of brain regions in the pain matrix. CG, Cingulate cortex; INS, insula; PoG, postcentral gyrus; Tha, thalamus. **(B)** Comparison of network efficiency of pain matrix. **(C)** Comparison of network local efficiency of pain matrix. **(D)** Brain regions with significant nodal efficiency differences between groups in pain matrix (FDR corrected). ^∗^*p* < 0.05, ^∗∗^*p* < 0.01, ^∗∗∗^*p* < 0.001.

**FIGURE 3:**
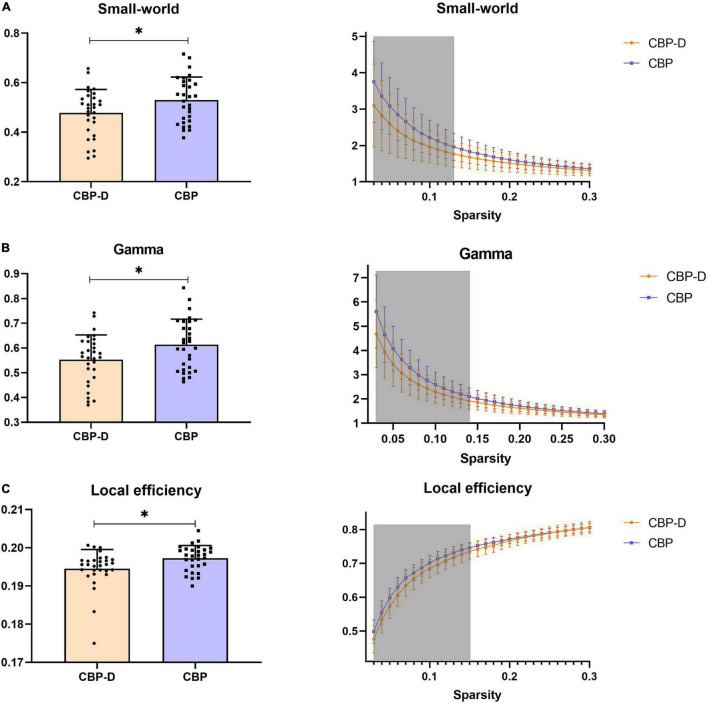
Inter group comparison of topological properties of whole brain. **(A)** The left column is the group comparison of the AUC of the small-world property under all sparsity thresholds. The right column is the specific results of the comparison between groups of small-world property under all sparsity thresholds. **(B)** The left column is the group comparison of the AUC of the Gamma property under all sparsity thresholds. The right column is the specific results of the comparison between groups of Gamma property under all sparsity thresholds. **(C)** The left column is the group comparison of the AUC of the local efficiency property under all sparsity thresholds. The right column is the specific results of the comparison between groups of local efficiency property under all sparsity thresholds. Shaded areas represent sparsity thresholds with significant inter-group differences. ^∗^*p* < 0.05.

Subjects in the CBP-D group had significantly lower nodal efficiency values in the left ventral posterior insula (VPI) (one of the brain regions of the pain matrix) after multiple comparison correction (*t* = −3.581, *p* = 0.0007) and had lower network efficiency values (*t* = −2.758, *p* = 0.008) and network local efficiency values (*t* = −2.475, *p* = 0.017) in the pre-determined pain matrix (Figure 2 and Table 3). The results of the graph theory analyses showed that the AUC of small-world property (*t* = −2.175, *p* = 0.034), gamma (*t* = −2.332, *p* = 0.023, see Supplementary Material for the detailed definition), and local efficiency (*t* = −2.461, *p* = 0.017) in the CBP-D group were significantly lower, but the global efficiency was not included (Figure 3 and Table 3).

**TABLE 3:** Inter group comparison of topological metrics.

	CBP-D	CBP	*t*-value	*P*-value
**Pain-matrix topological metrics**				
Nodal efficiency (VPI)	0.121 ± 0.027	0.141 ± 0.015	−3.581	<0.001
Network efficiency	0.132 ± 0.01	0.138 ± 0.007	−2.758	0.008
Network local efficiency	0.208 ± 0.01	0.213 ± 0.006	−2.475	0.017
**Topological metrics**				
Small-world	0.478 ± 0.095	0.530 ± 0.092	−2.175	0.034
Gamma	0.553 ± 0.01	0.614 ± 0.103	−2.332	0.023
Local efficiency	0.195 ± 0.005	0.197 ± 0.003	−2.461	0.017
Global efficiency	0.128 ± 0.008	0.131 ± 0.005	−1.769	0.083

VPI, ventral posterior insula.

**FIGURE 4:**
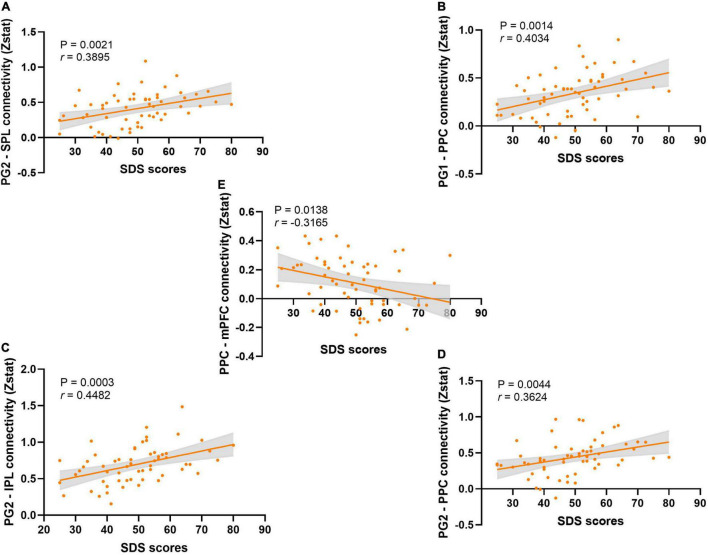
The correlation analysis between SDS scores and the functional connectivity. **(A)** The correlation analysis between the SDS scores and the PG2-SPL connectivity. **(B)** The correlation analysis between the SDS scores and the PG1-PPC connectivity. **(C)** The correlation analysis between the SDS scores and the PG2-IPL connectivity. **(D)** The correlation analysis between the SDS scores and the PG2-PPC connectivity. **(E)** The correlation analysis between the SDS scores and the PPC-mPFC connectivity.

The correlation analysis results (all *p*-FDR < 0.05, Figure 4) showed that the SDS scores were significantly positively correlated with PG2-SPL connectivity (*r* = 0.3895, *p* = 0.0021), PG2-PPC connectivity (*r* = 0.3624, *p* = 0.0044), PG1-PPC connectivity (*r* = 0.4034, *p* = 0.0014), and PG2-IPL connectivity (*r* = 0.4482, *p* = 0.0003). The SDS scores were also showed significantly negatively correlated with PPC-mPFC connectivity (*r* = −0.3165, *p* = 0.0138). At the same time, there was no significant correlation between pain intensity and FC (Supplementary Figure 3). Similarly, the analysis of covariance did not find that pain intensity had an effect on the difference of FC results between groups (Supplementary Table 2). After multiple comparison, we also found that there was no significant correlation between pain duration and FC, and between pain duration and SDS scores (Supplementary Figure 5, all *p*-FDR > 0.05). We also carried out the correlation analysis of the results of the graph theory analyses with SDS scores and pain intensity, respectively. The results revealed that the most results of the graph theory analyses were significantly negatively correlated with SDS scores (Supplementary Figure 4), but no significant correlation was found with pain intensity.

### Mediation analyses

**FIGURE 5:**
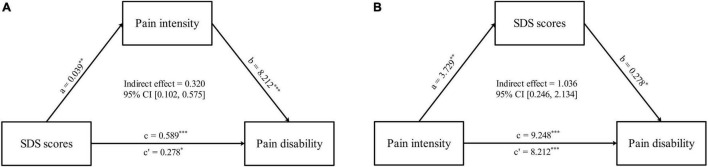
Comorbid depressive symptoms-pain intensity-pain disabilities associations. **(A)** The mediation analyses between SDS scores (*X*) and PDI scores (*Y*), with pain intensity as the mediators (M). **(B)** The mediation analyses between pain intensity (*X*) and PDI scores (*Y*), with SDS scores as the mediators (M). ^∗^*p* < 0.05, ^∗∗^*p* < 0.01, ^∗∗∗^*p* < 0.001.

Based on the results of correlation analysis, further mediation analyses demonstrated that pain intensity had a mediating effect on the relationship between SDS scores and PDI scores (indirect effect = 0.320, standard error [SE] = 0.119, 95% CI = [0.102, 0.575]). Likewise, the SDS scores mediated the relationship between pain intensity and PDI scores (indirect effect = 1.036, standard error [SE] = 0.483, 95% CI = [0.246, 2.134]) (Figure 5 and Table 4). No mediating effect of FC and the topological properties of the pain matrix on the relationship between SDS scores and pain-related clinical features was found.

**TABLE 4:** Detailed results of mediation analysis.

	Effect	SE	*t*-value	*P*-value	95% confidence interval	
					
					LLCI	ULCI
**The mediation analysis between SDS (*X*) and pain disability (*Y*)**						
Total effect	0.598	0.142	4.212	<0.001	0.314	0.882
Direct effect	0.278	0.108	2.575	0.013	0.062	0.494
Indirect effect	0.320	0.119			0.102	0.575
**The mediation analysis between pain intensity (*X*) and pain disability (*Y*)**						
Total effect	9.248	1.021	9.057	<0.001	7.204	11.292
Direct effect	8.212	1.055	7.787	<0.001	6.101	10.324
Indirect effect	1.036	0.483			0.246	2.134

SE, standard error.

## Discussion

Some researchers have studied the relationship between chronic pain and depression, but most of these studies are controlled by patients and healthy individuals (Bär et al., 2007; Bilek et al., 2019; Letzen and Boissoneault, 2020). The pain or depression induced by the experiment has a very short impact on the subjects, so it can only be a preliminary study of this interaction (Wiech and Tracey, 2009). This study found that the pain intensity scores and PDI scores in patients of CBP with depressive symptoms were significantly higher than those without depressive symptoms. Moreover, SDS scores were significantly correlated with the pain intensity scores and PDI scores. Follow-up mediation analysis then found that comorbid depressive symptoms could act as a mediator of the relationship between pain intensity and PDI scores, while the pain intensity could act as a mediator of the relationship between comorbid depressive symptoms and PDI scores. These results indicated that, similar to previous studies (Li, 2021), depressive symptoms do affect the clinical characteristics associated with chronic pain. After determining the interaction, we next made two assumptions about the relationship between CBP and depression: (1) CBP and depression accelerate disease progression by affecting the neural basis of each other and (2) CBP and depression fundamentally share some of their neurological underpinnings.

With the development of the graph network theory in neuroimaging, we can explore the topological properties of complex networks. As a complex network, the brain has the characteristics of high information transmission efficiency and high anti-interference ability (Biswal et al., 2010). Previous studies on healthy subjects (Chen and Hu, 2019) and some diseases, such as rheumatoid arthritis (Schrepf et al., 2018) and ischemic leukoaraiosis (Zhu et al., 2020), have been carried out in detail by using the graph theory method, and brilliant achievements have been obtained from the macro aspects of brain work efficiency, which provides a theoretical basis for better study of brain function. In partial accordance with our initial hypothesis, our results showed that the network efficiency and local efficiency of the pain matrix were lower in the CBP-D group. In this regard, the pain matrix network efficiency was significantly negatively correlated with SDS scores, while both topological properties were not correlated with pain intensity. Global efficiency and local efficiency focus on measuring the efficiency of information exchange between remote brain regions and between local networks, respectively (Rubinov and Sporns, 2010). Therefore, according to the research results and the definition of each topological property, we can reckon that comorbid depressive symptoms have led to the disconnection and instability of the FC of the pain matrix in patients with CBP. Our study also found several whole-brain topological properties were lower (uncorrected) in the CBP-D group and were also negatively correlated (uncorrected) with SDS scores but not with pain intensity. These findings demonstrated that co-morbid depressive symptoms can indeed exacerbate impaired function of the pain matrix network. However, subsequent mediation analyses did not find that these significantly different topological properties and FC mediate the interaction of depressive symptoms and CBP, which was inconsistent with our initial hypothesis. Up to this, the first hypothesis mentioned above is then not valid, that is, depressive symptoms cannot exacerbate CBP (at least the pain intensity) by affecting the neural basis of pain.

After identifying depressive symptoms can exacerbate functional impairment of the pain matrix, we next analyzed which brain regions and which functions of the pain matrix are specifically impaired by depressive symptoms in patients with CBP. We found that the nodal efficiency in the VPI showed significant between-group differences (FDR corrected), and the FC results also showed significant between-group differences in the FC of several brain regions of the pain matrix, including the mPFC, SPL, IPL, and PPC, and both this topological property and the FC values of these brain regions were significantly correlated with SDS scores (all FDR corrected) but not with pain intensity.

Previous studies have revealed that enhancing the basal neuronal activity of the prefrontal cortex (PFC) enhances its modulation of nociception, whereas patients with chronic pain show reduced neuronal projections from the PFC to the periaqueductal gray matter, decreased PFC glutamate levels, and reduced PFC gray matter density. Our study found that compared with the CBP group, the CBP-D group showed a significantly decreased FC pathway between the left mPFC and the right postcentral gyrus. This suggested that comorbid depressive symptoms aggravate the ability of pain regulation in patients with CBP. However, this FC pathway was significantly negatively correlated with SDS scores but not with pain intensity. It is commonly believed that the diminished prefrontal cortex activity is accompanied by complementary changes in brain regions associated with pain (such as the thalamus and primary somatosensory cortex) when negative emotions regulate pain (Wiech and Tracey, 2009), and a possible reasons for this phenomenon of depressive symptoms regulating pain may imply a partial overlap in the neural basis of the two disorders (Wiech and Tracey, 2009), for instance, the descending pain modulation pathway (Michaelides and Zis, 2019). Since research has shown that monoamine transmitters, such as serotonin and norepinephrine, play a role in reducing pain by acting on this modulation pathway (the PFC is the cortical center of the descending pain modulation pathway), the most commonly prescribed antidepressants (selective serotonin and norepinephrine reuptake inhibitors) are also used to treat neuropathic pain (Ossipov and Morimura, 2014; Sheng and Liu, 2017). These research advances, as well as the results of the present study, remind us that the brain regions in which functional activity was significantly correlated with SDS scores (rather than pain intensity) may be part of the common neural basis for depression and CBP. This implies the possibility that the second hypothesis we mentioned above is valid, that is, CBP and depressive symptoms fundamentally share some of their neurological underpinnings. Not coincidentally, chronic pain and mood disorders appear to share many other neuroendocrine and neurotransmitter changes, including substance P, gamma-aminobutyric acid, glutamate, and dopamine (Yalcin and Barrot, 2014; Pinheiro et al., 2015). In rats with neuropathic pain, microinjections of morphine into the endogenous opioid circuits can induce dopamine release from the nucleus accumbens while relieving pain (LaGraize and Borzan, 2006). In addition, pretreatment of the nucleus accumbens with a non-selective dopamine receptor blocker blocked the action of morphine on the endogenous opioid circuits to relieve pain aversion, and the opioid activity in the ACC is sufficient and necessary to maintain the increase in dopamine (Navratilova et al., 2015). The dopamine system is unique in the brain. Many depressive symptoms, such as a loss of pleasure and excitement, are directly tied to malfunction of the dopamine system (Eshel and Tian, 2016; Grace, 2016). The interaction between the dopamine system and endogenous opioid circuits can also be regarded as the commonality of pain and depression in neural pathways. Consequently, all these findings can give corresponding support to our research hypothesis.

Our study also found that, compared with the CBP group, there was an enhancement of FC in some parietal brain regions in the CBP-D group, including the SPL, IPL, and PPC. These brain regions are all related to attention, spatial discrimination, and awareness (Teixeira et al., 2014). They are also central to several classical brain networks, involving the ventral and dorsal attention networks, as well as the executive control networks (Smith et al., 2009; Fiebelkorn and Kastner, 2020). The role of the parietal cortex in pain processing has not been clearly clarified, but some researchers believe that it plays a role in maintaining attention to pain and spatial discrimination of noxious stimuli (Carlsson et al., 2006; Oshiro et al., 2007). It was found that the activity of the PPC kept pace with the abnormal increase of central sensitivity of pain (Seifert et al., 2010). According to the experiment, the researchers speculated that this was due to the abnormal increase of spatial attention to harmful stimuli. In addition, a new study has found that stimulation of some areas of the parietal cortex can reduce depression scores (Hou et al., 2021). The increased FC of the parietal cortex in this study is related to depression, indicating that the increased function of the parietal cortex caused by comorbid depression in this study may enhance the attention bias of patients to pain stimuli and enhance the perception of pain. This may also be one of the reasons for the higher pain intensity in patients with comorbid depression and pain.

Previous studies have shown that posterior insula activity can characterize pain to some degree (Mazzola, 2009) and that its increased activity contributes to pain perception control (Tran, 2010), while pain control is impaired in patients with posterior insula lesions (Starr et al., 2009). Our results revealed a significantly lower nodal efficiency in VPI in the CBP-D group compared to the CBP group, and this nodal efficiency was significantly negatively correlated with SDS scores. This suggests that the control of pain representations may be impaired in the presence of depressive symptoms.

Even if our study found some evidence that comorbid depressive symptoms can aggravate the functional changes of the pain matrix in patients with CBP, we must point out some shortcomings of this study to avoid them in the future. Firstly, due to the limited study time and subject population, our sample size is relatively small, which may lead to some evidence not being found. Secondly, anxiety symptoms and some other pain-related clinical characteristics may act as confounders or mediators to influence the study results and need to be included in subsequent in-depth studies in the future. Thirdly, we did not conduct hierarchical analysis on the duration of pain, which is a deficiency of this manuscript, and we hope to include it in the study in the future. Fourthly, excessive inter-individual variation in pain duration is also one of the limitations of this study, and we will analyze subgroups with excessive range of pain duration in future studies. Fifthly, healthy subjects were not included in this study.

## Conclusion

This study draws a preliminary conclusion that some brain regions of the pain matrix may be the common neural basis of depression and CBP. Comorbid depressive symptoms do interact with CBP, but depression does not aggravate pain intensity by directly affecting the function of the pain matrix. These results can enrich the theoretical system of chronic pain affected by different comorbid symptoms. Moreover, we hope that our research can stimulate more humanistic care for patients with chronic pain, and pay more attention to the recovery of psychosocial function.

## Data Availability Statement

The original contributions presented in this study are included in the article/Supplementary Material, further inquiries can be directed to the corresponding authors.

## Ethics statement

The studies involving human participants were reviewed and approved by Zhujiang Hospital of Southern Medical University and Guangdong Provincial Hospital of Chinese medicine. The patients/participants provided their written informed consent to participate in this study.

## Author contributions

All authors had full access to all the data in the study and took responsibility for the integrity of the data and the accuracy of the data analysis. GZ and WW: conceptualization. GZ, JM, WRL, XW, and WW: methodology. GZ, JM, WRL, XZ, and YH: investigation. JM, HZ, and KW: formal Analysis. QC and WL: experimental technology and equipment support. GZ, WRL, HZ, and WL: resources. JM, WRL, and XZ: data curation. GZ, JM, and XZ: writing – original draft. WP, SY, QC, and WL: writing review. QC, WL, and WW: supervision. HZ and WW: funding acquisition.

## Conflict of Interest

The authors declare that the research was conducted in the absence of any commercial or financial relationships that could be construed as a potential conflict of interest.

## Publisher’s Note

All claims expressed in this article are solely those of the authors and do not necessarily represent those of their affiliated organizations, or those of the publisher, the editors and the reviewers. Any product that may be evaluated in this article, or claim that may be made by its manufacturer, is not guaranteed or endorsed by the publisher.

## Footnotes

1
http://www.fil.ion.ucl.ac.uk/spm

2
http://www.nitrc.org/projects/gretna/

3
http://www.afhayes.com

## Abbreviations

fMRI: functional magnetic resonance imaging
SDS: self-rating depression scale
CBP: chronic back pain
CBP-D: chronic back pain with comorbid depressive symptoms
VAS: visual analog scale
PDI: pain disability index
ROI: region of interest
FC: functional connectivity
AI: anterior insula
ACC: anterior cingulate cortex
PG: postcentral gyrus
PPC: posterior parietal cortex
IPL: inferior parietal lobule
SPL: superior parietal lobule
mPFC: medial prefrontal cortex
VPI: ventral posterior insula.
