# Supplementary material for: Comorbid depressive symptoms can aggravate the functional changes of the pain matrix in patients with chronic back pain: A resting-state fMRI study
Source: Front Aging Neurosci. 2022 Jul 18;14:935242. doi: 10.3389/fnagi.2022.935242 (PMC9340779; doi:10.3389/fnagi.2022.935242)
Supplement: Supplementary file 1 [file Data_Sheet_1.docx]

Supplementary Materials

**MATERIALS AND METHODS**

**Data preprocessing**

The preprocessing of the fMRI data was carried out in the DPABI (Yan, Wang, Zuo, & Zang, 2016) toolbox based on Statistical Parametric Mapping (SPM12) software (www.fil.ion.ucl.ac.uk/spm). This procedure included data format conversion, discarding the first 10 volumes, slice timing correction, realignment, nuisance covariates regression (including Friston’s 24 motion parameters calculated from the six original motion parameters (Friston, 1996), white matter signals, cerebrospinal fluid signals, and movement artifact), spatial normalization to the Montreal Neurological Institute (MNI) stereotactic space, smoothing, linear de-trending and filtering. The effect of removing the first 10 time points was to eliminate the non-equilibrium effects of magnetization and the maladjustment of the subject. In addition to adding 24 head movement parameters as covariates to the linear regression, we also implemented other measures to minimize the impact of head movement on the data: censoring and scrubbing. We also applied a 3 mm translation and 3° rotation threshold for excessive head movement. The images of each subject were resampled at a resolution of 3 mm × 3 mm × 3 mm. The normalized functional images were smoothed spatially using a 6 mm full width at half maximum (FWHM) Gaussian kernel. Finally, we conducted linear de-trending to reduce the influence of low-frequency drift and filtering to retain the low-frequency band (0.01-0.08 Hz).

**Graph Theory Analyses**

Global efficiency measures the efficiency of information transmission between nodes in the whole brain network (Achard & Bullmore, 2007). Global efficiency is defined as the reciprocal of the average shortest path length (*L_p_*) of the whole-brain network. The path length is defined as the edges throughout all possible paths from nodes i to j in the graph. Therefore, the global efficiency for the brain (*E_global_*) is:

$$E_{global}=\frac{1}{N(N-1)}\sum_{i, j, i\neq j} \frac{1}{d_{ij}}$$

where N is the number of nodes and d_ij_ is the shortest path length from node i to node j.

Local efficiency is defined as the average global efficiency of all the subnetwork *G_i_* formed by the interaction of each neighbor of node i (ranges from 1 to 246) (Latora & Marchiori, 2001). Therefore, the local efficiency (*E_local_*) is:

$$E_{local}\left( i \right)=E_{global}\left( G_{i} \right)$$

Nodal local efficiency is defined as the global efficiency of a subnetwork *G_i_* formed by the interaction of each neighbor of node i (ranges from 1 to 246) (Latora & Marchiori, 2001).

Nodal efficiency is defined as the reciprocal of the average path length from node i to all other nodes in the network (Achard & Bullmore, 2007). Therefore, the nodal efficiency of node i *(E_nodal_i_*) is:

$$E_{nodal\_i}=\frac{1}{(N-1)}\sum_{j, j\neq i} \frac{1}{d_{ij}}$$

where N is the number of nodes and d_ij_ is the shortest path length from node i to node j. In this work, we used the Human Brainnetome Atlas with 246 brain regions of interest (ROIs), thus the number of N is 246.

The small-world network represents an optimal network that can balance the integration and segregation of information (Watts & Strogatz, 1998). Here, we need to first introduce another metric associated with the small-world network: the clustering coefficient (*C_p_*). The clustering coefficient is defined as the ratio of the edges that exist between the neighbor nodes of node i and the maximum number of possible edges. The *C_p_* measures the anti-interference ability of the communication between nodes in a local network. Compared with a random network, a small-world network has the characteristics of higher *C_p_* and approximately equivalent *L_p_*:

$$\gamma=\frac{C_{p}}{C_{p}^{rand}}>1 and \lambda=\frac{L_{p}}{L_{p}^{rand}}\approx1$$

Therefore, in the small-world network, the σ (σ = γ/λ, γ = Gamma, λ = Lambda), which measures the small-world network, should be greater than 1.

**Sample Size Calculation**

The sample size of this study was calculated using Gpower software (Gpower 3.1, http://www.gpower.hhu.de/). The alpha was set to 0.05 and the power was set to 0.8. We used the pre-experiment pain matrix network efficiency results as the primary outcome to calculate the effect size (Cohen's d), and the results was 0.6868028. We input the above three parameters in Gpower software, and the final calculated sample size was 27 in each group. Considering the possible 10% data quality control in each group, our final sample size was 30 in each group.

| Label ID | Regions | Brodmann area  And Modified cyto-architectonic | MNI | | |
| --- | --- | --- | --- | --- | --- |
|  |  |  | X | Y | Z |
| 155 | 'Postcentral Gyrus' | 'area 1/2/3' | -50 | -16 | 43 |
| 156 | 'Postcentral Gyrus' | 'area 1/2/3' | 50 | -14 | 44 |
| 157 | 'Postcentral Gyrus' | 'area 1/2/3' | -56 | -14 | 16 |
| 158 | 'Postcentral Gyrus' | 'area 1/2/3' | 56 | -10 | 15 |
| 159 | 'Postcentral Gyrus' | 'area 2' | -46 | -30 | 50 |
| 160 | 'Postcentral Gyrus' | 'area 2' | 48 | -24 | 48 |
| 161 | 'Postcentral Gyrus' | 'area1/2/3(trunk region)' | -21 | -35 | 68 |
| 162 | 'Postcentral Gyrus' | 'area1/2/3(trunk region)' | 20 | -33 | 69 |
| 163 | 'Caudodorsal Posterior Insula' | 'hypergranular insula' | -36 | -20 | 10 |
| 164 | 'Caudodorsal Posterior Insula' | 'hypergranular insula' | 37 | -18 | 8 |
| 169 | 'Ventral Posterior Insula' | 'ventral granular insula' | -38 | -4 | -9 |
| 170 | 'Ventral Posterior Insula' | 'ventral granular insula' | 39 | -2 | -9 |
| 171 | 'Rostrodorsal Posterior Insula' | 'dorsal granular insula' | -38 | -8 | 8 |
| 172 | 'Rostrodorsal Posterior Insula' | 'dorsal granular insula' | 39 | -7 | 8 |
| 179 | 'Cingulate Gyrus' | 'pregenual area 32' | -6 | 34 | 21 |
| 180 | 'Cingulate Gyrus' | 'pregenual area 32' | 5 | 28 | 27 |
| 183 | 'Cingulate Gyrus' | 'caudodorsal area 24' | -5 | 7 | 37 |
| 184 | 'Cingulate Gyrus' | 'caudodorsal area 24' | 4 | 6 | 38 |
| 187 | 'Cingulate Gyrus' | 'subgenual area 32' | -4 | 39 | -2 |
| 188 | 'Cingulate Gyrus' | 'subgenual area 32' | 5 | 41 | 6 |
| 235 | 'Thalamus' | 'sensory thalamus' | -18 | -23 | 4 |
| 236 | 'Thalamus' | 'sensory thalamus' | 18 | -22 | 3 |

**Table S1.** The region information of the prior defined mask

**Table S2.** The effect of the pain intensity gap between groups on FC values (Covariance Analysis)

| FC values | Mean Square | F value | *P* value |
| --- | --- | --- | --- |
| **PG1-PPC** |  |  |  |
| Group*pain intensity | 0.053 | 1.305 | 0.258 |
| Cor | < 0.001 | 0.011 | 0.915 |
| **PG2-PPC** |  |  |  |
| Group*pain intensity | 0.043 | 0.869 | 0.355 |
| Cor | 0.012 | 0.241 | 0.625 |
| **PG2-IPL** |  |  |  |
| Group*pain intensity | 0.015 | 0.285 | 0.595 |
| Cor | 0.008 | 0.148 | 0.702 |
| **PG2-SPL** |  |  |  |
| Group*pain intensity | 0.043 | 0.913 | 0.344 |
| Cor | 0.010 | 0.213 | 0.647 |
| **PPC-mPFC** |  |  |  |
| Group*pain intensity | < 0.001 | 0.019 | 0.891 |
| Cor | 0.002 | 0.082 | 0.776 |

Group*pain intensity represents the hypothesis test of whether the slopes of two regression lines are equal. P > 0.05 means that the condition of parallel regression lines is met, and covariance analysis can be performed. Cor represents hypothesis test of whether pain intensity was a covariate of FC results difference between groups. P > 0.05 means that the pain intensity has no effect on the inter group difference of FC results.

**Figure S1.** The brain map of seed-to-whole-brain functional connectivity analysis results. The upper left number represents the MNI coordinate slice position. Warm tone (red) represents brain regions with increased functional connectivity strength between groups, while cold tone (blue) represents brain regions with decreased functional connectivity between groups.

**Figure S2.** The correlation analysis between the clinical characteristics. (A) The correlation analysis between the pain intensity and the PDI scores. (B) The correlation analysis between the SDS scores and the PDI scores. (C) The correlation analysis between the pain intensity and the SDS scores.

**Figure S3.** The correlation analysis between pain intensity and the functional connectivity. (A) The correlation analysis between the pain intensity and the PG2-SPL connectivity. (B) The correlation analysis between the pain intensity and the PG1-PPC connectivity. (C) The correlation analysis between the pain intensity and the PG2-IPL connectivity. (D) The correlation analysis between the pain intensity and the PG2-PPC connectivity. (E) The correlation analysis between the pain intensity and the PPC-mPFC connectivity.

**Figure S4.** The correlation analysis between SDS scores and the results of graph theory analyses. (A) The correlation analysis between the SDS scores and the nodal (VPL) efficiency. (B) The correlation analysis between the SDS scores and the pain matrix efficiency. (C) The correlation analysis between the SDS scores and the pain matrix local efficiency. (D) The correlation analysis between the SDS scores and the Gamma. (E) The correlation analysis between the SDS scores and the local efficiency. (F) The correlation analysis between the SDS scores and the small-world.

**Figure S5.** The correlation analysis between pain duration (months) and the functional connectivity, and between pain duration (months) and the SDS scores (all *p*-FDR > 0.05). (A) The correlation analysis between the pain duration (months) and the PG1-PPC connectivity. (B) The correlation analysis between the pain duration (months) and the PG2-IPL connectivity. (C) The correlation analysis between the pain duration (months) and the PG2-PPC connectivity. (D) The correlation analysis between the pain duration (months) and the PG2-SPL connectivity. (E) The correlation analysis between the pain duration (months) and the PPC-mPFC connectivity. (F) The correlation analysis between the pain duration (months) and the SDS scores.

**References:**

Achard, S., & Bullmore, E. (2007). Efficiency and cost of economical brain functional networks. *PLoS computational biology, 3*(2), e17. doi:10.1371/journal.pcbi.0030017

Friston, K. (1996). Movement-related effects in fMRI time-series. *Magnetic resonance in medicine, 35*(3), 346-355. doi:10.1002/mrm.1910350312

Latora, V., & Marchiori, M. (2001). Efficient behavior of small-world networks. *Physical review letters, 87*(19), 198701. doi:10.1103/PhysRevLett.87.198701

Watts, D., & Strogatz, S. (1998). Collective dynamics of 'small-world' networks. *Nature, 393*(6684), 440-442. doi:10.1038/30918

Yan, C., Wang, X., Zuo, X., & Zang, Y. (2016). DPABI: Data Processing & Analysis for (Resting-State) Brain Imaging. *Neuroinformatics, 14*(3), 339-351. doi:10.1007/s12021-016-9299-4
