# Supplementary figures and images for: Comorbid depressive symptoms can aggravate the functional changes of the pain matrix in patients with chronic back pain: A resting-state fMRI study
Source: Front Aging Neurosci. 2022 Jul 18;14:935242. doi: 10.3389/fnagi.2022.935242 (PMC9340779; doi:10.3389/fnagi.2022.935242)

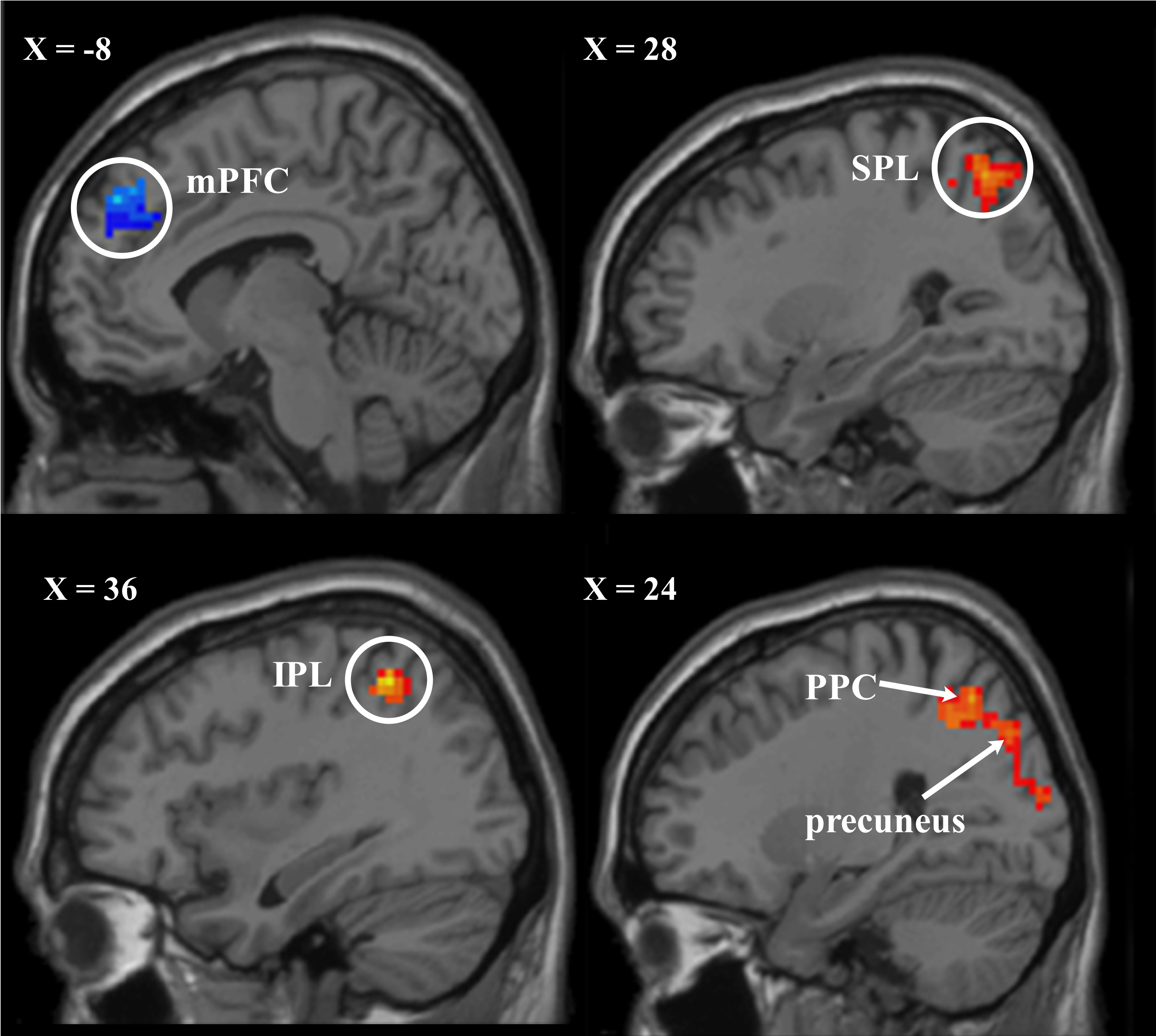

Supplement: Supplementary file 2 [file Image_1.TIF]

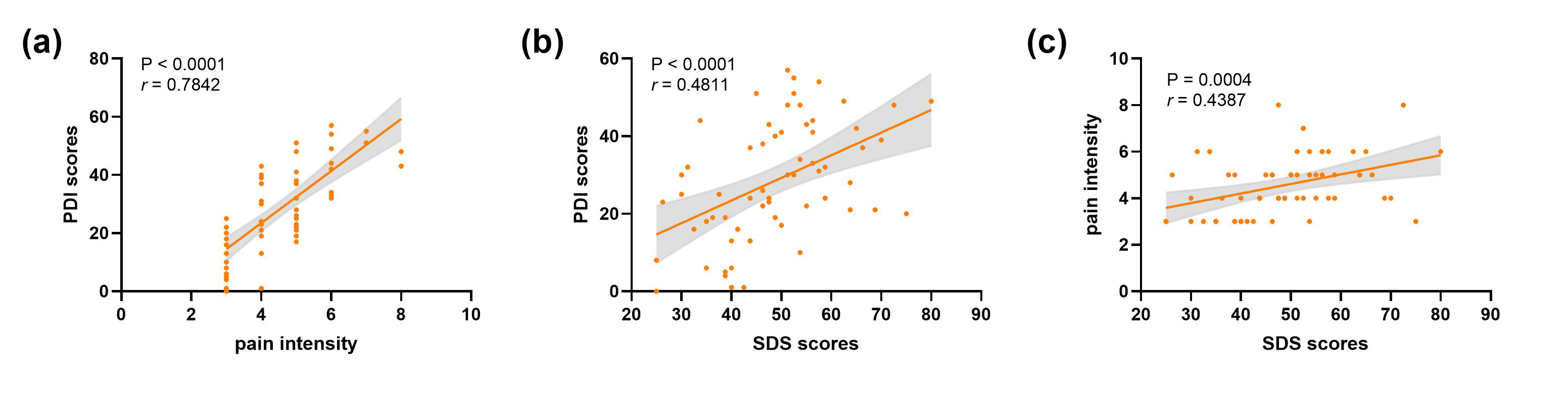

Supplement: Supplementary file 3 [file Image_2.TIF]

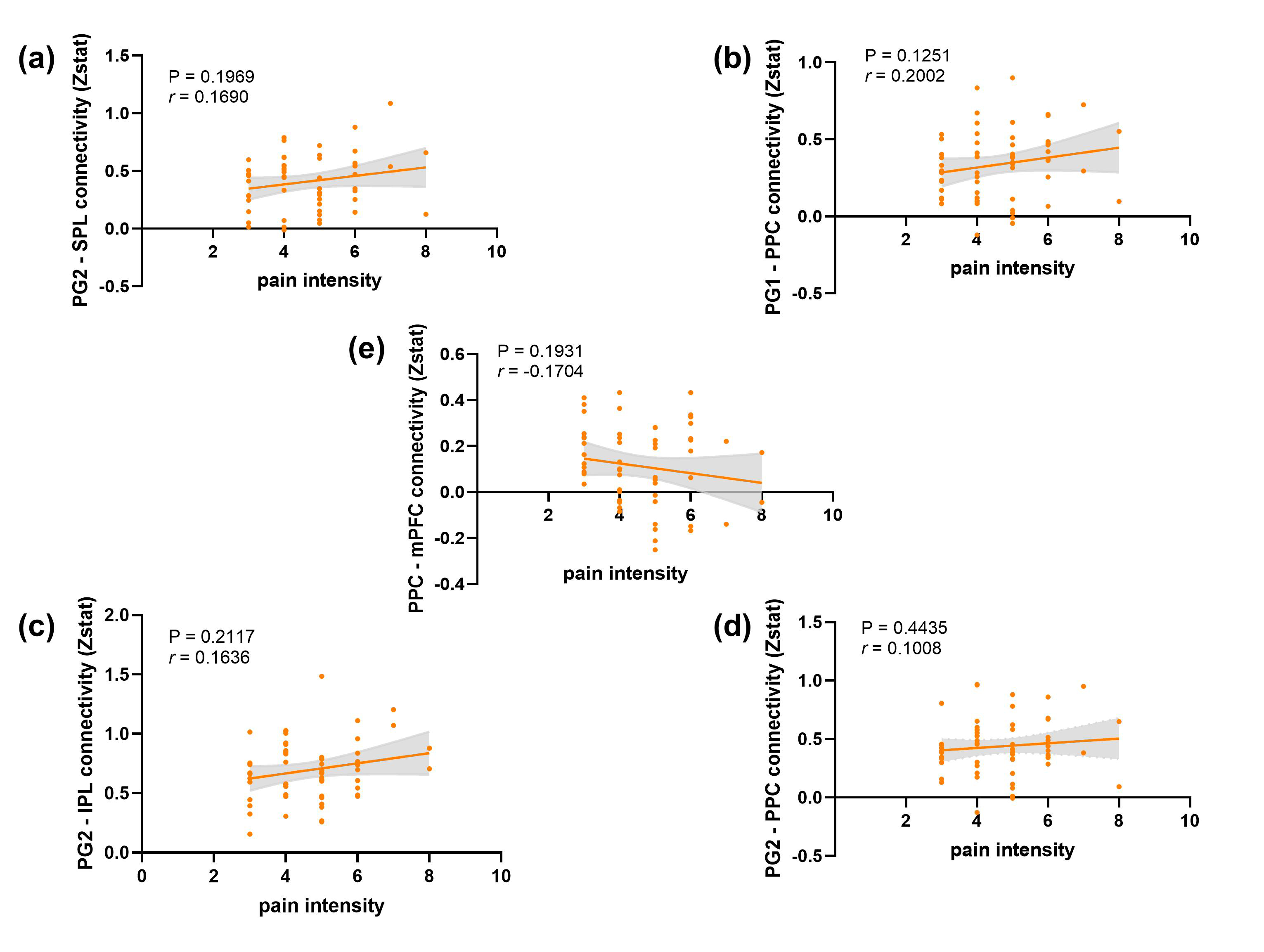

Supplement: Supplementary file 4 [file Image_3.TIF]

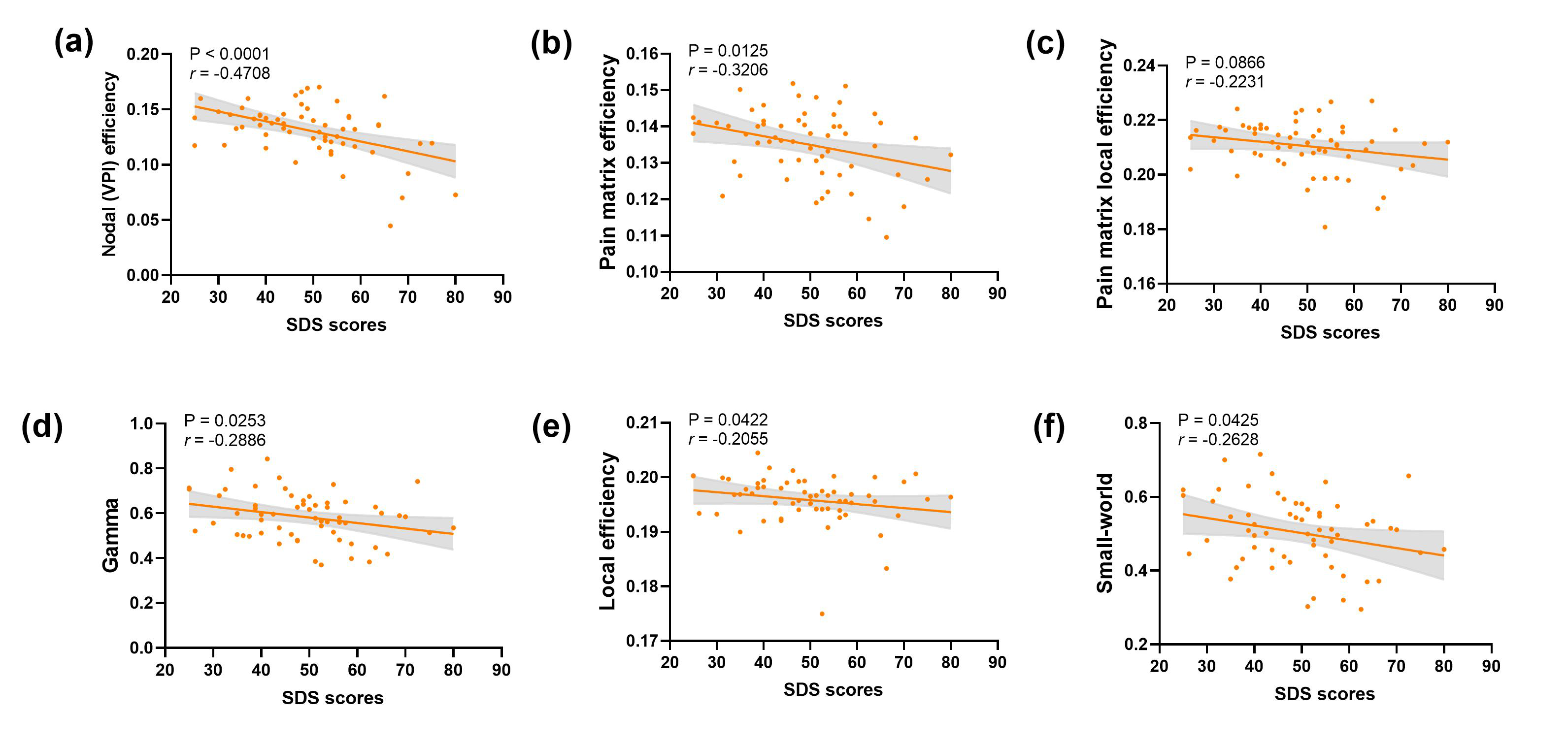

Supplement: Supplementary file 5 [file Image_4.TIF]

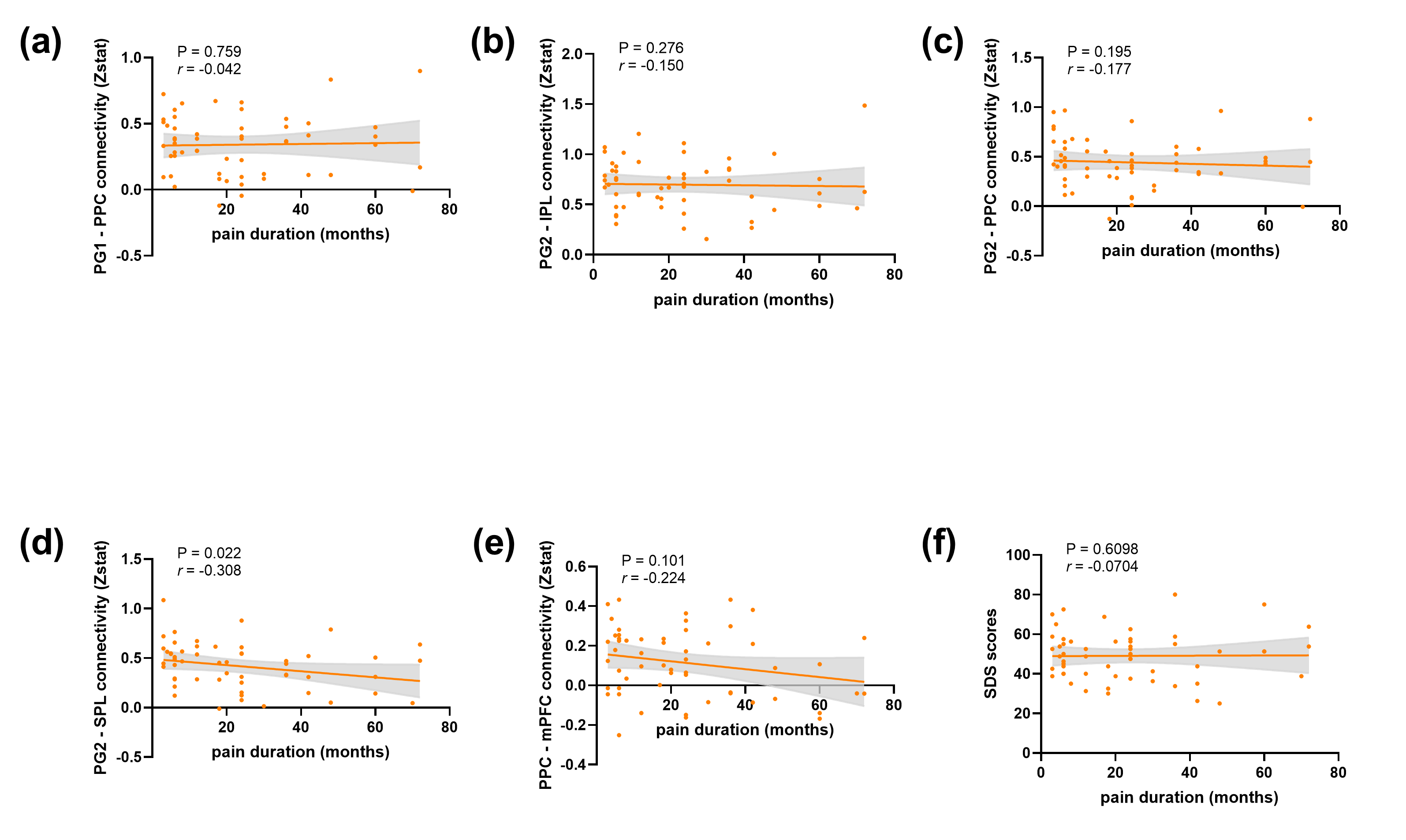

Supplement: Supplementary file 6 [file Image_5.TIF]
